# A large number of variants of *Corynebacterium diphtheriae c*ausing diphtheria outbreaks in Indonesia, 2010–2017

**DOI:** 10.7717/peerj.21718

**Published:** 2026-09-28

**Authors:** Sunarno Sunarno, Sundari Nursofiah, Novi Amalia, Yudi Hartoyo, Nelly Puspandari, Lisa Andriani Lienggonegoro, Ratih Dian Saraswati, Dwi Febriyana, Tati Febrianti, Khariri Khariri, Yuni Rukminiati, Kambang Sariadji, Masri Sembiring Maha, Yustinus Maladan, Ariyani Noviantari, Christina Safira Whinie Lestari, Masagus Zainuri, Pretty Multihartina, Rahadian Pratama, Siswanto Siswanto

**Affiliations:** 1 Center for Biomedical Research, National Research and Innovation Agency (BRIN), Bogor, Indonesia; 2 Centre for Research and Development of Biomedical and Basic Health of Technology, National Institute of Health Research and Development, Jakarta, Indonesia; 3 National Health Biology Laboratory, Ministry of Health, Jakarta, Indonesia; 4 Health Policy Agency, Ministry of Health, Jakarta, Indonesia; 5 Eijkman Research Center for Molecular Biology, National Research and Innovation Agency (BRIN), Bogor, Indonesia; 6 Department of Biochemistry, Faculty of Mathematics and Natural Sciences, IPB University, Bogor, West Java, Indonesia

**Keywords:** *Corynebacterium diphtheriae*, Diphtheria, Indonesia, Disease outbreaks, CgMLST

## Abstract

**Background:**

Several methods exist for the molecular typing of diphtheria-causing bacteria, including core genome multilocus sequence typing (cgMLST). This study aims to provide an overview of the sequence types, virulence factors, and molecular resistances of Corynebacterium diphtheriae (*C. Diphtheriae*) in different regions of Indonesia between 2010–2017 to predict diseases transmission patterns and evaluate control measures .

**Methods:**

A total of 89 archived isolates of *C. diphtheriae* collected between 2010 and 2017 from 10 provinces in Indonesia were used as test samples. DNA extraction and whole genome sequencing (using the QIAamp DNA Minikit and Illumina MiSeq, respectively) followed standard protocols. Bioinformatic analysis was performed using a pipeline that included ‘diphtOscan’.

**Results:**

Seventy out of eighty-nine samples qualified for analysis. Fifteen sequence types (STs) were identified: ST377 (24.3%), ST534 (44.3%) and others 31.4%. Approximately 50% of isolates predicted as Gravis biovar, based on *spu*A gene presence; two isolates were predicted as Belfanti due to *nar*G gene absence. The Gravis biovar includes isolates with ST105, ST123, and ST534. The cgMLST analysis revealed 14 sublineages corresponding to sequence types; we also identified 33 genetic clusters (GC) and 20 clonal groups among the analyzed samples. Notable GC such as GC1197, GC1199, and GC9 were found across provinces and islands. Isolates with ST141/GC75 shared similarity with Malaysian isolate; those with ST377/GC9 showed resemblance to Indian or European Union counterparts. The antibiotic resistance genes (ARGs) including *cmx*, *tet*W and *tet*O, *sul*1, *aad, aph-*Ib *, aph-*Ia *, aph-*Id, *dfr*A*,* were identified in most isolates. In contrast, *msr*(D)*, mef*(A)*,* which encode erythromycin resistance via *erm*X, were found in only two isolates. One isolate harbored *pbp*2, responsible for penicillin resistance. Notably, almost all ST377 isolates exhibited aminoglycoside resistance; conversely, the single non-ST377 isolate showed this trait as well.

**Conclusion:**

This study demonstrates that Indonesia’s 2010–2017 diphtheria outbreaks were caused by multiple strains, with ST534 and ST377 being the most prevalent. GC similarity suggests a link or disease transmission between various outbreaks across different regions /islands/countries and periods. ARGs were predominantly associated with phenicol, tetracycline, and sulfonamides resistances; only a few isolates exhibited ARGs linked to penicillin and erythromycin. Our analysis revealed correlations between STs and biovar or resistance profiles. We recommend to implement whole genome sequencing (WGS) for diphtheria surveillance in Indonesia.

## Introduction

Diphtheria is a significant global health problem that affects low- and middle-income countries. The circulation of non-toxigenic diphtheria-causing bacteria strains also poses challenges in developed nations (Xiaoli et al., 2020). Persistent annual outbreaks have been reported in India, Indonesia, and other countries worldwide, underscoring the need for sustained control efforts. Indonesia has been a consistent high-burden country for diphtheria, despite achieving an 80% diphtheria, tetanus, and pertussis (DTP)-3 vaccination rate (Kementerian Kesehatan, 2018b). The WHO reported significant numbers of cases in the region during 2010–2017 (excluding 2015), diphtheria cases were consistently high: 432, 806, 1,192, 775, 430, 342, and 954 reported in Indonesia during this period (World Health Organization, 2024). Indonesia has historically ranked among the top four countries with the highest diphtheria case numbers globally.

Data from the Indonesian Ministry of Health indicate a widespread occurrence of diphtheria across almost all provinces, spanning from Aceh Province in the West to Papua Province in the East. The government declared outbreaks in several provinces between 2010 and 2017. **Initial outbreak period (2010–2013)**: In 2010, a total of 385 cases were reported by the ministry, predominantly among children under five years old (Kementerian Kesehatan, 2011). The number of cases surged in 2012 and 2013, with 1,192 cases recorded across 18 provinces (case fatality rate (CFR): 6.38%) and 778 cases reported across 19 provinces (CFR: 5.01%), respectively (Kementerian Kesehatan, 2014; Kementerian Kesehatan, 2015). **Period of decline (2014–2016)**: Cases decreased significantly to 396 in 19 provinces (CFR: 4.04%), 252 cases in 13 provinces (CFR: 1.98%), and 415 cases in 20 provinces (CFR: 5.8%) during this period (Kementerian Kesehatan, 2016; Kementerian Kesehatan, 2017; Kementerian Kesehatan, 2018c). **Resurgence (2017-2018)**: The number of cases increased substantially due to an outbreak centered around Indonesia’s capital city and surrounding areas in 2017. By that year, a total of 954 cases were reported across 30 provinces (CFR: 4.61%) (Kementerian Kesehatan RI, 2018c). In 2018, the number of cases peaked at 1,386 across all but five provinces (Yogyakarta, West Nusa Tenggara, East Nusa Tenggara, Central Sulawesi, and West Papua), with a corresponding decline in CFR to 2.09% (Kementerian Kesehatan, 2019). Although province-level analysis shows no significant inverse correlation between case numbers and vaccination coverage, individual-level data showed that most Indonesian cases either lacked DTP vaccination or had received only partial vaccination (Harapan et al., 2019; Husada et al., 2024).

The primary causative agent of diphtheria is *Corynebacterium diphtheriae*, which exhibit four distinct biotypes: Gravis, Mitis, Intermedius, and Belfanti. Previous studies have reclassified several isolates of the Belfanti type as new species, including *Corynebacterium belfantii* and *Corynebacterium rouxii* (Prygiel et al., 2022; Schlez et al., 2021). Diphtheria caused by *C. ulcerans* is more frequently reported in highly developed countries with high vaccination coverage, such as the United Kingdom, Germany, Japan, etc. In contrast to other regions, there are no documented human cases of diphtheria caused by *C. pseudotuberculosis*, despite its ability to harbour the *tox* gene (Bernard et al., 2019; Brunello, 2019; Gower et al., 2020; Wagner et al., 2012). Recent classifications have reclassified some isolates of *C. pseudotuberculosis* as *Corynebacterium silvaticum* (Viana et al., 2023). Diphtheria-causing bacteria are classified based on their capacity for toxin production, with two primary categories: toxigenic and non-toxigenic species. The former harbors the tox gene, synthesizes diphtheria toxin, and is associated with disease manifestations. In fact, some non-toxigenic strains (NTTB) retain the tox gene but have lost their ability to produce toxin phenotypically (Doyle et al., 2017; Prygiel et al., 2022).

The genotypic characteristics of bacteria causing diphtheria can be used for epidemiological investigations and interventions, including mapping disease transmission/spread, planning interventions, and evaluating public health measures. Molecular typing is an approach to identifying bacterial genotypes (Ruppitsch, 2016). Several methods exist for molecular typing of diphtheria-causing bacteria; multilocus sequence typing (MLST) is the most widely used method (Bolt et al., 2010; Graham et al., 2023; Sekizuka et al., 2020). An advanced MLST analysis using genomic information, known as core genome multilocus sequence typing (cgMLST), offers higher resolution and discriminatory power (Ghanem et al., 2018). The cgMLST approach have been applied in several studies of Corynebacterium diphtheriae (*C. Diphtheriae*) (Seth-Smith & Egli, 2019) and was used to investigate a recent outbreak in Yemen (Badell et al., 2021), which revealed connections between isolates from Somalia, Eritrea, Saudi Arabia, Poland and Venezuela (Strauss et al., 2021; Wołkowicz, Zacharczuk & Zasada, 2023). In this study, we employed the cgMLST approach to analyze *C. diphtheriae* strains responsible for outbreaks in various regions of Indonesia between 2010 and 2017. We conducted cgMLST analysis to predict associations between cases or outbreaks and correlated bacterial virulence and antibiotic resistance with sequence type. It is important to evaluate the management of diphtheria case and the prevention of disease transmission.

## Materials & Methods

### Samples

A total of 89 archived isolates of *C. diphtheriae* from cases and close contacts were used as test samples. The close contacts were defined as people who had contact with diphtheria case intensively, in a period 10 days before onset and 2 days after therapy according to the national guideline for diphtheria surveillance in Indonesia (Kementerian Kesehatan, 2018a). Samples were collected from different regions of Indonesia in the period 2010–2017, with some samples already analyzed by using seven alleles-based MLST in a previous study (Sunarno, Rukminiati & Saraswati, 2020). The samples were stored in TSB+15% glycerol media at −70 °C. Samples were recultured on blood agar plates and incubated overnight at 37 °C. Colonies were harvested and placed in tubes containing 0.5 ml aquadest for DNA extraction. All laboratory works was conducted at the Prof. Dr. Sri Oemijati Research Laboratory for Infectious Diseases in Jakarta (currently known as National Health Biology Laboratory), one of the national reference laboratories for diphtheria in Indonesia.

### DNA extraction

DNA isolation was performed using a commercial kit, the QIAamp DNA Mini Kit (Qiagen, Hilden, Germany), according to the manufacturer’s protocol with minor modifications. DNA samples were stored in 50 µl molecular water. The purity/quality of DNA was adjusted based on an absorbance value of 260/280 nm, while DNA quantification was measured using the Qubit^®^3.0 Fluorometer (Thermo Fisher Scientific, Waltham, MA, USA) and the dsDNA HS Assay Kit (Thermo Fisher Scientific, Waltham, MA, USA). The quality and quantity of the DNA had to meet the requirements for the Illumina MiSeq platform.

### DNA sequencing

DNA sequencing was performed using a next-generation sequencing (NGS) approach with the MiSeq platform (Illumina, San Diego, CA USA). The DNA libraries were prepared using the Nextera XT DNA Flex Library Prep Kit (Illumina, San Diego, USA). DNA libraries were adjusted using the Agilent 4200 TapeStation system and gel electrophoresis. Whole genome sequencing (WGS) was performed using the Illumina MiSeq platform following these steps: denaturing the libraries, diluting the libraries, preparing the optional PhiX control, loading the libraries onto the reagent cartridge, and setting up the sequencing run.

### Bioinformatics and statistical analysis

The Bioinformatic process begins with quality control of the sequencing reads. Reads were processed to remove reads with a quality score below 20, a minimum length of 100 bp, and 5 bps were trimmed at the 5′ and 3′. Filtering and trimming of reads were performed using Fastp v0.23.4 (Chen et al., 2018) with the parameter ‘*-f 5 -q 20 -l*100 − *c* − *w 5 -b 290 -B 290 –detect_adapter_for_pe*’. High-quality reads were assembled using SPAdes v3.15.5 (Prjibelski et al., 2020) with the parameter ‘*-k 21,33,55,77 –isolate -t 5 -m 20*’ and then polished using Pilon v1.24 (Walker et al., 2014) to correct misassembled bases. The polished draft assemblies were evaluated using QUAST v5.2.0 (Gurevich et al., 2013) and used for sequence typing analysis. To evaluate the assembly completeness, ‘checkm’ (Parks et al., 2015) was used with default parameter.

Multilocus sequence typing (MLST) and core genome MLST (cgMLST) analysis were performed with polished assemblies. Both MLST and cgMLST analysis were performed simultaneously using ‘diphtOscan’ with an updated database from May 22nd, 2024 (Hennart et al., 2023). The parameter used in ‘diphtOscan’ was ‘*–mlst –tox –resistance_virulence –tree*’. The result of ‘diphtOscan’ was further processed using iTOL v6 (Letunic & Bork, 2024) to generate a phylogenetic tree based on cgMLST, complete with associated information such as sequence typing, *tox* gene, biovar type and common resistance genes. To investigate the geographic distribution of *C. diphtheriae* and frequent sequence types (ST), geographic analysis was performed using Microreact v258 (Argimón et al., 2016) with the newick file from the ‘diphtOscan’ result.

### Ethical statement

This study was part of the routine national surveillance of diphtheria by the Ministry of Health. The samples were archive isolates of *C. diphtheriae* with no individual data (completely anonymous). Therefore, neither informed consent nor ethical clearance was required, as stated by the Ethics Committee of the National Institute of Health Research and Development, Ministry of Health, Indonesia, in ethics statement No. LB.02.01/2/KE.216/2017.

## Results

### Sample characteristics

A total 70 out of 89 raw genomic data of bacterial isolates passed quality control filters. Isolates were collected over an 8-year period from 10 provinces in Indonesia, as depicted in Fig. 1). A majority (78.9%, *n* = 56) of the samples originated from three regions: Jakarta, Banten, and West Java, where accounted for approximately 75% of diphtheria cases reported nationwide during this time frame (Kementerian Kesehatan, 2018b). Bioinformatic analysis using diphtOscan pipeline revealed strong correspondence between two samples (1,593 and 53) and *Corynebacterium belfantii*. Thirty-three isolates were predicted as Gravis biovar based on *spuA* gene presence (Supplement 1), whereas remaining isolates were classified as Mitis or Intermedius biovars. A high proportion of *tox-*positive strains, accounting for approximately 92.8% (*n* = 65) of all isolates examined, was observed. Interestingly, a single isolate lacking the toxin gene (*tox-*negative) belonged to *C. diphtheriae* and originated from an individual suspected of having contracted diphtheria; in contrast, other isolates were obtained from close contacts.

**Figure 1:**
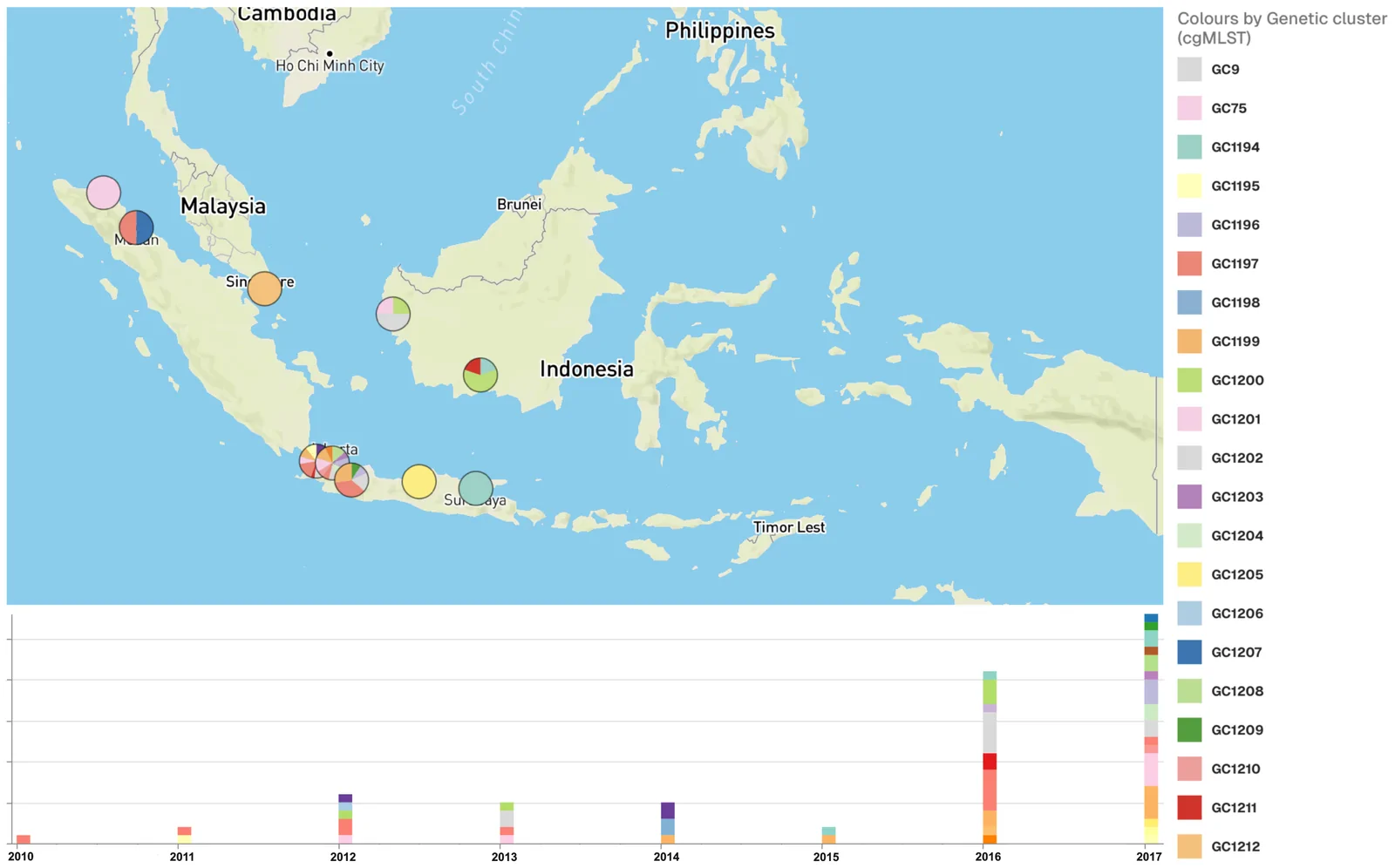
Geographic, collecting time, and phylogenetic distribution of isolates by MLST profile. This analysis was performed with Microreact v258, interactive result available at: https://microreact.org/project/tBArjSmyGd2nHEmErSyi2R-cdiphtheriae (Argimón et al., 2016) (doi: 10.1099/mgen.0.000093).

### Whole genome sequence reads quality

Following quality control using fastp, we retained 70 samples with high-quality reads for downstream analysis. The average Phred score was above Q20. The subsequent assembly and polishing steps yield a draft genome assembly of approximately 2.4 Mbps in size, comprising contigs ranging from under 1,000 bps.

### Core genome molecular typing

Fifteen STs were identified from the 70 isolates based on MLST analysis of seven alleles. The prevalence of ST534 and 377 is evident, accounting for approximately 69% of all samples. Both ST534 and 377 were consistently present in samples collected over several years from different regions. In contrast, some STs were found to be unique to individual isolates. The distribution of STs spanned various regions within Indonesia (Fig. 1). Further analysis using the core genome MLST (cgMLST) approach revealed that 14 sublineages (SL) highly correlated with seven alleles-based ST (Fig. 2), consistent with a previous study by Hennart et al. (2023). A single isolate exhibited identical sublineage but distinct sequence type (ST302 and ST1097), suggesting relatedness (Figs. 1 and 2). Additionally, we identified 20 clonal groups (ClG) and 33 genetic clusters (GC), which several GC have not been reported before.

**Figure 2:**
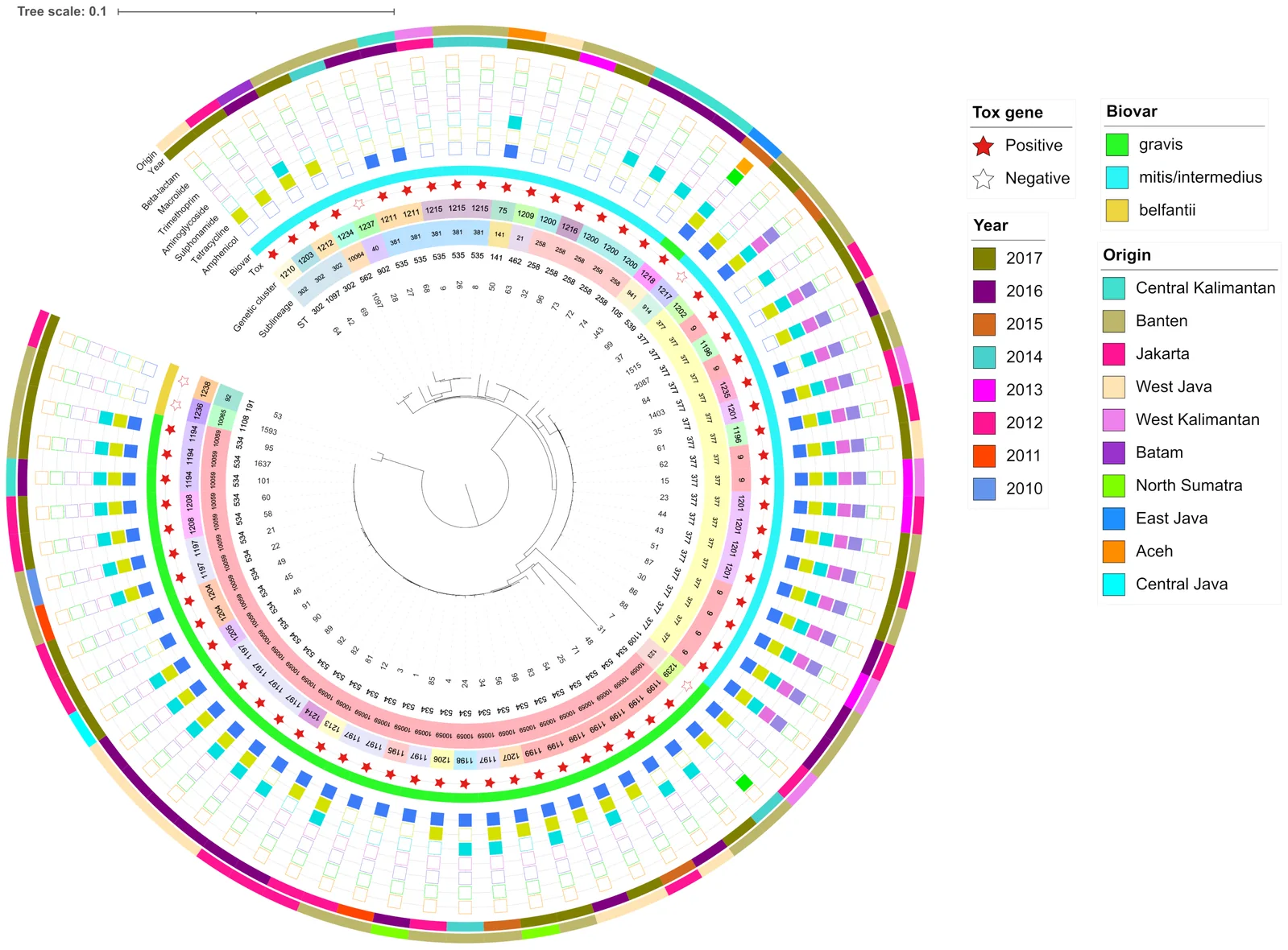
Phylogenetic tree based on Core genome MLST analysis from 70 diphtheria isolates. Tree was annotated with ST, sublineage, genetic cluster, *tox* gene, biovar prediction and antimicrobial resistance genes (ARGs), included the region sources and collection time. Annotation was performed using iTOL v7.

GC exhibits a precise relationship between isolates compared to ST and SL in predicting disease transmission as reported by Hennart et al. (2023). This study identified 12 GC from ST534 and five GC from ST377 as the most common ST associated with disease outbreaks. Figure 1 illustrates the distribution of these strains. GC9 (ST377), first detected in West Kalimantan in 2013, was subsequently found in neighboring regions Banten, West Java, and Jakarta by 2016. In 2017, GC9 isolates were identified in both Banten and West Java. Another ST377-associated cluster, GC1201, emerged in a diphtheria case from West Kalimantan (Borneo Island) in 2012. Although no information is available on the travel or contact history of this individual with other cases, subsequent isolates were detected in Jakarta by 2013 and again in both Jakarta and Banten by 2017. Similar spatial–temporal spread of multiple GCs was observed across various provinces and islands within a relatively short period. Unfortunately, direct contact between individual cases could not be established due to lack of available information on travel or contact history. Furthermore, cgMLST analysis demonstrated high discriminatory power in typing *Corynebacterium* species by identifying several subclades within each major clade (ST534 and ST377).

DiphtOscan analysis revealed correlations between ST and biovar predictions in this study. A strong association was observed between specific STs (ST105, ST123 and ST534) and the presence of the *spu* A gene, a marker for Gravis biovar. Conversely, Two STs (ST191 and ST1108) were found lacking the *narG* gene which encodes nitrate reductase (Hennart et al., 2020), suggesting they may belong to Belfanti biovar as classified by DiphtOscan (Supplement 1). By DiphtOscan, three *tox* gene variants identified in 65 *tox* positive isolates, including allele 2 (18.5%), allele 3 (53.8%), and allele 8 (27.7%). Almost all isolates tested positive for the *tox* gene*,* however, a correlation between STs and *tox* gene presence was challenging to establish due to this high prevalence of *tox* positivity. Notably, all isolates with ST1108 and ST191 were found to be *tox* negative.

### Antimicrobial resistance

Analysis of antimicrobial resistance gene (ARGs) identified the presence of multiple resistance genes. The presence of ARGs in the isolate genomes indicate resistance to several antibiotics, including amphenicol (*cmx*), tetracycline (*tet*), sulphonamide (*sul1*), aminoglycoside (*aad, aph-Ib, aph-Ia, aph-Id*), and trimethoprim (*dfrA*). In contrast, the presence of *msr* (D), *mef* (A)*, erm* (X) genes (encode macrolide/lincosamide/streptogramin resistant) and *pbp* 2 (encode beta-lactams resistant) were limited (Fig. 2, Supplement 1). Based on ARGs analysis, two isolates showed evidence of resistance to macrolide/lincosamide/streptogramin antibiotics (related to erythromycin resistance), samples 31 and J43. In addition, sample J43 also exhibited possible beta-lactam resistance (related to penicillin resistance).

To some extent, the patterns of antimicrobial resistance appear to be associated with STs. Almost all isolates belonging to ST377 (except sample 1515), regardless of year isolated, indicated resistance to aminoglycosides. In contrast, only one non-ST377 isolate (ST105) was indicated resistant to aminoglycoside (sample J43). Sample 1515 did not exhibit trimethoprim resistance, whereas the remaining ST377 isolates showed possible resistance to this antibiotic. Similarly, both ST377 and ST105 exhibited potential resistance to quaternary ammonium-based disinfectants (*qacEdelta*) (Supplement 1).

## Discussion

This study provided an overview of the variation in characteristics among *C. diphtheriae* and Corynebacterium belfantii (*C. Belfantii*) isolates from Indonesia between 2010 and 2017 (Fig. 1). Isolates were obtained from diphtheria cases and close contacts across ten provinces out of 38 in Indonesia. According to a Ministry of Health report (Kementerian Kesehatan, 2018b), the majority (88.1%) of the total 954 reported diphtheria cases in 2017 originated from these ten provinces. Geographically, the samples were collected from three large islands (Sumatra, Java, Kalimantan) and one smaller island. Approximately two-thirds (65.1%) of isolates were obtained between 2016 and 2017, a period marked by an increase in diphtheria cases within Jakarta and surrounding areas (Banten and West Java), as reported by Ministry of Health, (Kementerian Kesehatan, 2018b). Unfortunately, samples from East Java (the province with the most diphtheria cases in Indonesia) were very limited (only one isolate). In addition, the proportion of samples analyzed is quite small compared to the total number of cases, so they may not represent all diphtheria cases in Indonesia. This limitation is acknowledged as a major concern in this study. Isolates from cases before 2010 were not included in this study. A group of researchers previously collected isolates from a hospital in Jakarta and used the isolates globally, but we did not have access to them (Rappuoli, Perugini & Ratti, 1987; Rockhill et al., 1982).

The *C. diphtheriae* biovar Mitis was predicted as the most common cause of diphtheria in Indonesia in this study (Supplement 1), consistent with reports from other countries (Benamrouche et al., 2016; Mohd Khalid et al., 2019). Interestingly, Gravis biovar was found in approximately 50% of samples. This finding presents an uncommon distribution of Gravis biovar compared to other countries. This study reports on two Belfanti biovar (reported as *C. belfantii)* collected from close contact individuals. In general, Belfanti biovar is non-toxigenic and rarely identified as a cause of diphtheria (Farfour et al., 2013; Pivot et al., 2019). Furthermore, the predominance of *tox* positive strains found in this study is similar to what has been previously reported in developing countries (Bhagat et al., 2015), while non-toxigenic strains are tipically isolated from developed countries (Dangel et al., 2018; Public Health England, 2018). Similar to our previous study, non-toxigenic *tox* gene bearing (NTTB) strains were not found in this study based on *tox* gene sequence variants. NTTB strains are commonly reported in developed countries. A previous study reported an NTTB strain isolated from an Australian traveler who returned from Indonesia, but it was not associated with a diphtheria case (Doyle et al., 2017). According to the WHO guidelines, the diphtheria case was confirmed by the presence of a toxigenic *Corynebacterium* strain in the clinical sample (World Health Organization, 2018). Although the non-toxigenic strains are not considered causative agent of diphtheria, they still possess the potential to cause another fatal disease such as endocarditis (Muttaiyah et al., 2011). Moreover, non-toxigenic strain can be converted to toxigenic when *tox* gene-carrying phage lysogenizing those strains (Groman & Memmer, 1958).

In this study, we used the core genome information (cgMLST) approach, which offers more precision than MLST based on seven alleles. Utilizing the diphtOscan pipeline, classifications based on both MLST and cgMLST were conducted. According to MLST, 15 STs out of 70 identified samples were confirmed in this study (ST/total sample (n) ratio: 19.7%). This number is relatively high, although lower than in report from Spain, where 28 STs were identified from 39 samples (ST/n: 71.8%) (Hoefer et al., 2021) and Austria, where 34 STs were identified from 57 isolates (ST/n: 69.6%) (Schaeffer et al., 2021). Of the 15 identified STs, more than half of the isolates have not been reported from the other countries, including isolates with ST534, ST535, and ST539. ST535 and ST539 were not predominant, and even ST539 was only identified in one isolate. ST535 was found in two regions on two different islands and across different years. In contrast, ST534 appears to be the most frequent, especially in the Indonesian capital and surrounding areas, as previously reported (Sunarno, Rukminiati & Saraswati, 2020). Interestingly, ST534 also circulated in other areas, such as Central Java, which is adjacent to West Java and North Sumatra, which are on different islands than the other four areas. ST534 has been isolated almost every year except 2013, it is abundant in Indonesia. The ST534, including its 12 GC reported in this study has not been identified from any other country to date.

Another frequent variant was ST377, which was found in several regions and even included in three major islands. This variant was isolated from 2012 to 2017, except for 2015. Reports from other countries indicate that ST377 was previously found in India but was not the common variant (Sangal et al., 2017). Another country that reported ST377 was Spain, where it was identified in a six-year-old child, nine of his schoolmates and one family member of a school friend (Jané et al., 2018). Hoefer et al. (2025) stated that this ST377 is one of the frequent sequence types across countries. However, among the five GCs of ST377 identified in this study, there are several GCs that have not been reported from other countries, including GC1196, GC1201, GC1202, and GC1235. Based on the similarity of GC9 from ST377, some diphtheria cases in Indonesia are predicted to be closely related to diphtheria cases in other countries, such as India, the UK, and European Union countries. In this study, GC9 did not or had not yet carried the integron containing the *erm*X gene encoding resistance to macrolides, contrasting with isolates from India and the European Union (Hoefer et al., 2025).

There are five sequence types (STs) that match the types reported in neighboring countries, including ST141, ST302, ST462, ST562, and ST902. A search of the global database revealed that ST141 was isolated from Malaysia in 1981, 2003, and 2008. Another search also found GC similarity (GC75) between isolates with ST141 from Malaysia and Indonesia. Conversely, no GC similarity was found in several STs, such as ST302 (Malaysia and Australia), ST462, and ST902. ST302 was reported in the Philippines in 2016 and 2017 and was considered the frequent variant in the country. ST302 was previously isolated from German travelers who had traveled to Malaysia. Additionally, it was isolated from Australia in 2017 and 2018. ST462 was reported from Malaysia in 2016, while ST902 was isolated from Australia in 2020 and 2021. Notably, ST562 was reported from Singapore. Unfortunately, there is insufficient data, especially genetic cluster information on isolates of ST302 and ST562 reported from Philippines and Singapore to predict the relationship between this isolate and diphtheria cases in Indonesia (Lai et al., 2018; Mohd Khalid et al., 2019; Saito et al., 2020). This is important, considering that Singapore is often used as a destination country for treating patients from Indonesia. Given the similarity in sequence types, this suggests that transmission of diphtheria has occurred between Indonesia and its neighboring countries. However, this needs to be validated with epidemiological data or a comprehensive cgMLST analysis that include whole-genome sequences between the countries.

In this study, we identified distinct GCs in a main sequence type or sublineage, similar to those found in a previous study (Hennart et al., 2023). Genomic or genetic clusters (GCs) exhibited high precision genetic classification of *C. diphtheriae* isolates were consistent with recent disease transmission (Guglielmini et al., 2021). Therefore, genomic clusters appear more relevant than sublineages (SL) or sequence types (ST) for epidemiological investigation purposes (Hennart et al., 2023). However, this study finds it challenging to accurately predict disease transmission because only some of the bacterial isolates causing diphtheria in Indonesia were examined. Although there are numerous benefits to be gained from whole genome sequencing (Seth-Smith & Egli, 2019), Indonesia has not yet implemented it in diphtheria surveillance. In Indonesia’s diphtheria surveillance system, only laboratory-confirmed cases undergo testing, and not all isolates are subject to whole genome sequencing. Furthermore, the high circulation of diphtheria-causing bacteria in the environment or among carriers without causing clinical symptoms, particularly in endemic area, creates gaps in case connections (Hughes et al., 2015). The discovery of the same GC in an area with a significant time gap raises speculation that the strain continues to circulate in that area without causing disease. However, there is a possibility of disease transmission to other areas and, at some point, transmission back to the original area. This is evident in several genomic clusters in this study (Figs. 1 and 2).

The province of Banten and its surrounding areas are the primary locations where most diphtheria strains circulate, as indicated by the GC profiles obtained in this study. According to GC profiles, it is hypothesized that all cases of diphtheria on Borneo Island are closely related to cases of diphtheria on Java Island, particularly in the province of Banten. Some diphtheria cases on Sumatra Island are also suspected to be closely related to cases on Java Island, although there is no known connection between diphtheria cases in Aceh and Batam and those in Banten and its surrounding areas based on GC similarity. Similarly, diphtheria cases in Central Java and East Java show no direct connection to diphtheria cases in Banten and its surroundings based on GC similarity. However, based on SL and ST similarity, it is predicted that some different GC strains initially originated from the same cluster (Fig. 2).

Regarding antimicrobial resistance prediction, particular attention must be paid to resistance to penicillin and erythromycin, as both are the main antibiotics of first line for diphtheria therapy. In this study, we found that one isolate (1.4%) predicted to be resistance to penicillin based on the *pbp2* gene, whereas two (2.8%) indicated resistance to erythromycin based on *msr* (D), *mef* (A), and *erm* (X) genes (Fig. 2). A recent study utilizing a large global dataset of 976 deduplicated *C. diphtheriae* genome identified *pbp2m* in 34 (3.5%) isolates and *erm* (X) in 36 (3.7%) isolates (Hennart et al., 2023). Several previous studies conducted in Indonesia have reported higher non-susceptible *C. diphtheriae* to penicillin were classified as intermediate (Husada et al., 2019; Sariadji et al., 2018). This discrepancy highlights the need to investigate the resistance mechanism of *C. diphtheriae* to penicillin, particularly for isolates with intermediate susceptibility.

Finally, Guglielmini et al. (2021) and Hennart et al. (2023) conducted a global analysis utilizing hundreds to thousands of isolates from multiple countries, which resulted in the development of the newly created diphtOscan pipeline, with the aim of becoming a standard tool for diphtheria analysis employing the cgMLST approach. The diphtOscan facilitates harmonized characterization of bacteria causing diphtheria sublineages of concern (Hennart et al., 2023). In the future, disease surveillance for diphtheria could be performed in all countries based on whole genome sequence (WGS) data, as demonstrated by this study, which has shown that the WGS data can discover sequence typing profile, virulence factors, and gene resistance profiles.

## Conclusions

We concluded that the diphtheria outbreaks in Indonesia were caused by multiple strains with ST534 and ST377 being the most abundant variants. A connection was indicated between various diphtheria outbreaks in different regions, islands, and countries across different periods as revealed by cgMLST analysis based on genetic cluster similarity. The identification of the same genetic cluster across consecutive years within the same region suggests suboptimal prevention and control efforts in that area. Conversely, the detection of the same genetic cluster in different regions highlights the association between cases and underscores the need for more effective transmission chain interruption strategies. Our results also suggest that most antimicrobial resistance genes (ARGs) correspond to phenicol, tetracycline, and sulfonamides, while few ARGs correspond to penicillin and erythromycin. Furthermore, we found a correlation between STs and biovar or resistance profiles.

##  Supplemental Information

10.7717/peerj.21718/supp-1 Supplemental Information 1 The diphtOscan raw data

## Additional Information and Declarations

### Data Availability

The following information was supplied regarding data availability: The FASTQ sequence data is available at DDBJ: PRJDB12216
The sequence data is available at Institut Pasteur: ST-534 (MLST), ST-535 (MLST), ST-539 (MLST) - https://bigsdb.pasteur.fr/cgi-bin/bigsdb/bigsdb.pl?page=profileInfo&db=pubmlst_diphtheria_seqdef&scheme_id=3&profile_id=534
- https://bigsdb.pasteur.fr/cgi-bin/bigsdb/bigsdb.pl?page=profileInfo&db=pubmlst_diphtheria_seqdef&scheme_id=3&profile_id=535
- https://bigsdb.pasteur.fr/cgi-bin/bigsdb/bigsdb.pl?page=profileInfo&db=pubmlst_diphtheria_seqdef&scheme_id=3&profile_id=539
